# Increased body mass index and adjusted mortality in ICU patients with sepsis or septic shock: a systematic review and meta-analysis

**DOI:** 10.1186/s13054-016-1360-z

**Published:** 2016-06-15

**Authors:** Dominique J. Pepper, Junfeng Sun, Judith Welsh, Xizhong Cui, Anthony F. Suffredini, Peter Q. Eichacker

**Affiliations:** Critical Care Medicine Department, Clinical Center, National Institutes of Health, Clinical Center Building 10, Room 2C145, 10 Center Drive, Bethesda, MD 20892 USA; National Institutes of Health Library, Clinical Center, National Institutes of Health, Bethesda, MD 20892 USA

**Keywords:** Sepsis, Obesity, Overweight, Body mass index, Mortality, Meta-analysis

## Abstract

**Background:**

At least 25 % of adults admitted to intensive care units (ICU) in the United States have an overweight, obese or morbidly obese body mass index (BMI). The effect of BMI on adjusted mortality in adults requiring ICU treatment for sepsis is unclear. We performed a systematic review of adjusted all-cause mortality for underweight, overweight, obese and morbidly obese BMIs relative to normal BMI for adults admitted to the ICU with sepsis, severe sepsis, and septic shock.

**Method:**

PubMed, the Cochrane Library, and EMBASE electronic databases were searched through November 18, 2015, without language restrictions. We included studies that reported multivariate regression analyses for all-cause mortality using standard BMI categories for adults admitted to the ICU for sepsis, severe sepsis, and septic shock. Articles were selected by consensus among multiple reviewers. Electronic database searches yielded 10,312 articles, of which six were eligible. Data were extracted by one reviewer and then reviewed by three independent reviewers. For the meta-analyses performed, the adjusted odds ratios (aOR) of mortality were combined using a random-effects model. Risk of bias was assessed using the Newcastle-Ottawa quality assessment scale for cohort studies.

**Results:**

Four retrospective (n = 6609 patients) and two prospective (n = 556) studies met inclusion criteria. Compared to normal BMI, across five studies each, overweight or obese BMIs reduced the adjusted odds ratio (95 % CI) of mortality [aOR] [0.83 (0.75, 0.91) *p* < 0.001 and 0.82 (0.67, 0.99) *p* = 0.04, respectively] with low or moderate heterogeneity (I^2^ = 15.7 %, *p* = 0.31 and I^2^ = 53.0 %, *p* = 0.07, respectively). Across three studies each, morbidly obese BMI and underweight BMI did not alter aOR [0.90 (0.59, 1.39), *p* = 0.64; I^2^ = 43.3 %, *p* = 0.17; and 1.24 (0.79, 1.95), *p* = 0.35; I^2^ = 15.6 %, *p* = 0.31 respectively]. Only one study clearly defined how and when height and weight measurements were calculated. Site of underlying infection and illness severity may have favored overweight and obese BMIs.

**Conclusions:**

This is the first meta-analysis to show that overweight or obese BMIs reduce adjusted mortality in adults admitted to the ICU with sepsis, severe sepsis, or septic shock. More rigorous studies that address these limitations are needed to clarify the impact of BMI on sepsis ICU outcomes.

**Trial registration:**

PROSPERO International prospective register of systematic reviews 10.15124/CRD42014010556. Registered on July 11, 2014.

**Electronic supplementary material:**

The online version of this article (doi:10.1186/s13054-016-1360-z) contains supplementary material, which is available to authorized users.

## Background

At least 25 % of adults admitted to intensive care units (ICU) in the United States (US) [1–4] have overweight, obese, or morbidly obese body mass indices (BMIs), while bacterial sepsis [5–7] is commonly the cause for these admissions. Although an obese BMI reduces overall life expectancy [8, 9], it is unclear whether it also impacts the acute outcome of ICU patients in general, or with sepsis specifically [3, 4, 10]. While identifying such an association has important prognostic and therapeutic implications, this is difficult because an obese BMI is one of several variables potentially influencing ICU outcomes. Studies addressing this question provide conflicting and unclear results [11].

A recent analysis of seven studies [12] of septic patients found an obese BMI increased [13], decreased [14–16], or had no effect [17–19] on survival. However, two of the studies included non-ICU and ICU patients for whom the overall risk of death would have differed [13, 14]. Furthermore, one study included children and adults [18], and another study did not account for other baseline variables [19].

Based on the adverse effects of an obese BMI on long-term health, we hypothesized that an increased BMI would also worsen short-term outcomes in adult patients with sepsis requiring ICU care. To examine this question, we performed a meta-analysis of studies in adult patients admitted to the ICU (participants) and treated for sepsis, severe sepsis, or septic shock (interventions/exposures). We examined the effect of different BMI categories (comparisons) on mortality (outcome) after adjusting for other influential baseline variables.

## Materials and methods

### Literature search and study selection

We performed a systematic literature review using published guidelines and registered the planned meta-analysis July 11, 2014 [20–22] in PROSPERO (International prospective register of systematic reviews 10.15124/CRD42014010556). Using search terms listed in Additional file 1, three authors (D.J.P., J.W., and A.F.S.) identified relevant studies in the following databases from inception through November 18, 2015 and without language restrictions: MEDLINE, EMBASE, and the Cochrane Central Register of Controlled Trials (CENTRAL). Included studies were searched for additional references. Author consensus resolved uncertainty regarding study inclusion.

Studies meeting the following criteria were analyzed (Additional file 2): employed prospective or retrospective observational study designs (study design); enrolled adult patients (≥16 years old) admitted to the ICU (participants) and treated for sepsis, severe sepsis, or septic shock (intervention/exposures); compared mortality (outcome) in patients across two or more BMI categories (comparisons) [23]; and employed multivariate analysis to adjust the effect of elevated BMI on mortality with other baseline variables. Although the search strategy included types of illnesses associated with sepsis (e.g., pneumonia and influenza), only studies that enrolled patients based on accepted definitions for sepsis, severe sepsis, or septic shock [24–26] were included in the systematic review and meta-analysis [22]. A priori, studies of trauma, primary surgical conditions, or surgical interventions complicated by nosocomial infection, and studies available only as abstracts were excluded [22].

### Data extracted and outcomes examined

Data was extracted from included studies using a standardized tool (Additional file 3). Definitions of sepsis, severe sepsis, and septic shock employed in studies had to be consistent with recognized and accepted definitions [24–26]. Of note, all studies were conducted prior to the new guideline nomenclature for sepsis and septic shock. The definitions and nomenclature used in these prior studies were used in our study. Authors of included studies were contacted when data required clarification.

The primary outcome examined was the effect of BMI on the adjusted odds ratio of mortality, considered in the following hierarchy: ICU, hospital, 28-day, 30-day, or 60-day mortality. Outcomes are presented based on comparisons between patients with normal BMI (18.5 to <25 kg/m2) versus those with underweight (<18.5 kg/m2), overweight (25 to <30 kg/m2), obese (30 to <40 kg/m2), or morbidly obese (≥40 kg/m2) BMIs. Composite outcomes were not examined.

### Statistical analysis

For the meta-analyses performed, the adjusted odds ratios (aOR) of mortality were combined using a random-effects model [27]. If a study reported an adjusted hazard ratio (aHR) instead of an adjusted odds ratio, the aHR was converted to aOR using the observed normal BMI group mortality rate with the assumption of proportional hazard. For example, in the study by Wacharasint et al. [16], the 28-day mortality rate of the normal BMI group was used. When the estimated effect was based on a continuous measure of BMI (i.e., aOR of mortality when BMI increases by 1 kg/m2), the midpoint of the BMI category was used in the analysis (e.g., BMI of 22 kg/m2 used for normal BMI [18.5–25 kg/m2], BMI of 27.5 kg/m2 for overweight [25–30 kg/m2] and BMI of 35 kg/m2 for obesity [30–40 kg/m2]). Heterogeneity among studies was assessed statistically using the standard chi-square tests and I^2^ values [28]. Risk of bias of individual studies for outcomes was assessed using the Newcastle-Ottawa quality assessment scale for cohort studies (Additional file 4). Because only three to five studies were present in each BMI subgroup, publication bias could not be assessed. A priori, we combined obese BMI data from five studies with overweight BMI data from the one study without obese patients to strengthen our ability to detect an effect of moderately increased BMI on adjusted mortality. All analyses were performed using R (version 3.1.0) packages *meta* (version 4.1-0) [29, 30]. Two-sided *p* values ≤0.05 were considered significant. This review was prepared according to the PRISMA statement checklist (Additional file 5).

## Results

**Fig. 1:**
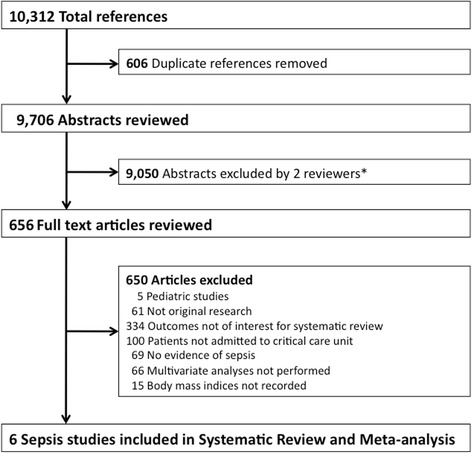
Study selection flow diagram. *Studies involving trauma, primary surgical conditions, or surgical interventions complicated by nosocomial infections, as well as studies of children, and those occurring outside the critical care unit were excluded

Our literature search identified 10,312 articles, 656 of which underwent full-text review and six of which met our inclusion criteria (Fig. 1). These six studies examined the effects of BMI on adjusted mortality in adult ICU patients with sepsis, severe sepsis, or septic shock [15–17, 31–33]. These six (n = 7165 patients) studies examined the effect of BMI on mortality (ICU, 28-day, 30-day, and 60-day in hospital mortality in one study each and hospital mortality in two studies) after adjusting for other baseline characteristics (Tables 1 and 2) [15–17, 31–33]. All six studies were included in our meta-analysis.

**Table 1:** Study characteristics

Author (Y)	Country	Study design	Study period (MM/YY)	Diagnostic criteria	Age, years Median (IQR or SD)	Male (%)	Outcome
Studies of sepsis, severe sepsis and septic shock^a^							
Sakr [31] (2008)	24 European countries^b^	Prospective cohort, multicenter	05/02–05/02	Septic shock [25]	NR	NR	Hospital mortality
Wurzinger [15] (2010)	Austria	Retrospective cohort, single center	01/03–12/08	Septic shock [25]	69 (14)	162 (54)	ICU mortality
Adamzik [32] (2011)	Germany	Prospective cohort, single center	NR	Severe sepsis [25]	57 (16)	90 (58)	30-day mortality
Arabia [17] (2013)	Canada, USA Saudi Arabia	Retrospective cohort, multicenter	/96–/08	Septic shock [25]	NR	1658 (58)	Hospital mortality
Wacharasint [16] (2013)	Australia, Canada, USA	Retrospective cohort, multicenter	07/01–04/06	Septic shock [25]	NR	449 (62)	28-day mortality
Sakr [33] (2015)	84 countries^c^	Retrospective worldwide audit	05/12–05/12	Sepsis [26]	NR	NR	60-day in-hospital mortality

*NR* not reported

^a^Definitions of sepsis, severe sepsis and septic shock in these studies included Bone et al. [25] and Vincent et al. [26]

^b^Austria, Belgium, Czech Republic, Denmark, Finland, France, Germany, Greece, Hungary, Ireland, Israel, Italy, Netherlands, Norway, Poland, Portugal, Romania, Serbia and Montenegro, Slovakia, Slovenia, Spain, Sweden, Switzerland, and United Kingdom

^c^List of 84 countries detailed in the following link: http://links.lww.com/CCM/B435

**Table 2:** Studies with multivariate analyses for mortality

						Baseline study characteristics		
Author (Y) (Total patients)	BMI studied (kg/m2)	Non-survivor/total	BMI	Age	Gender	Comorbid illnesses	Severity of acute illness	Other
Studies of sepsis, severe sepsis, and septic shock								
Sakr [31] (2008) (n = 431)	Underweight (<18.5)	NR/17^c^	NR	NR	NR	NR	SAPS II	NR
	Normal (18.5–24.9)	NR/179^c^						
	Overweight (25–29.9)	NR/148^c^						
	Obese (30–39.9)	NR/76^c^						
	Very obese (>40)	NR/11^c^						
Wurzinger [15] (2010) (n = 301)	Underweight (<18.5)	3/15	Yes^d^	Yes	Yes	COPD, HTN, DM, CRI, HO, heart disease	SAPS II^d^	Admission year, origin of sepsis
	Normal (18.5–24.9)	28/125						
	Overweight (25–29.9)	15/95						
	Obese (30–39.9)	4/66						
Adamzik [32] (2011) (n = 125)	Continuous BMI^a^	NR	Yes	Yes	Yes	Hemofiltration/dialysis	SAPS II, SOFA score	IL-6^d^, aquaporin 5 genotype^d^, CRP, serum aldosterone, plasma angiotensin II, procalcitonin
Arabi [17] (2013) (n = 2882)	Underweight (<18.5)^b^	121/196	Yes	Yes	Yes	ID, HF, COPD, DM, Elective surgery	APACHE II	Type and source of bacterial infection, type of sepsis interventions
	Normal (18.5–24.9)	580/1020						
	Overweight (25–29.9)^b^	444/816						
	Obese (30–39.9)	349/680						
	Morbidly obese (>40)	76/170						
Wacharasint [16] (2013) (n = 730)	Continuous BMI^a^	NR	Yes^d^	NR	Yes	DM	APACHE II^d^	Lung infection, fungal infection
Sakr [33] (2015) (n = 2696)	Underweight (<18.5)	NR	Yes	Yes	Yes	Comorbidities^e^	SAPS II, SOFA score	Type and source of admission, need for mechanical ventilation or renal replacement therapy at ICU admission, type of hospital, ICU specialty, total number of ICU patients in 2011, number of staffed ICU beds, gross national income of country
	Normal (18.5–24.9)	NR						
	Overweight (25–29.9)	NR						
	Obese (30–39.9)	NR						
	Very obese (>40)	NR						

*BMI* body mass index, *NR* not reported, *SAPS* Simplified Acute Physiology Score, *COPD* chronic obstructive pulmonary disease, *HTN* arterial hypertension, *DM* diabetes mellitus, *CRI* chronic renal insufficiency, *HO* hematologic/oncologic disease, *SOFA* Sepsis-related Organ Failure Assessment score, *IL-6* interleukin-6, *CRP* C-reactive protein, *ID* immunodeficiency, *HF* heart failure, cancer, heart disease, *APACHE* Acute Physiology and Chronic Health Evaluation, *ICU* intensive care unit

^a^Continuous BMI: regression model used with body mass index as a continuous variable

^b^Adjusted odds ratio for mortality not provided by study for this BMI category

^c^Numerator not reported

^d^Independently associated with mortality in multivariate analysis

^e^Comorbidities not listed in multivariate analysis

Of the six studies examining mortality, two were prospective; one a single-center study from Germany [32] and the other a multicenter one from 24 European countries, which included a subgroup of patients with septic shock [31]. The other four studies were all retrospective; a single-center study from Austria [15] and three multicenter studies, one from the US, Canada, and Saudi Arabia [17], one from the US, Canada, and Australia [16] and one from 730 ICUs from 84 countries [33]. In studies assessing mortality, the proportion of patients investigated from the populations of patients identified as having sepsis, severe sepsis, or septic shock varied from 33 to 94 % (Table 3). Three of these studies included patients with BMIs calculated only from weight and height measures [15–17] while two studies included patients with estimated BMIs [31, 32], and another did not report how BMI was determined [32]. Only one study reported that BMI was determined before ICU admission and before fluid therapy potentially altered this determination [16].

**Table 3:** Characteristics of body mass index (BMI) assessment

		BMI measurement		
Author (Y)	Study populations	Method	Timing	Number with BMI/total patients (%)
Studies of severe sepsis, and septic shock				
Sakr [31] (2008)	431 patients with septic shock from 2878 patients with BMIs recorded in a survey of 3147 critically ill patients from 198 European countries. The total number of patients with septic shock out of all 3147 patients was not reported	Based either on recorded weight and height or on provider’s clinical estimate	At ICU admission	2878/3147 (91 %)
Wurzinger [15] (2010)	301 (88 %) patients with BMIs recorded from 343 patients with septic shock admitted to a single ICU in Austria. A total of 2700 ICU admissions were screened to obtain the 343 patients with septic shock	Based on recorded weight and height	At ICU admission	301/343 (88 %)
Adamzik [32] (2011)	125 (81 %) patients with BMIs recorded from 154 patients with severe sepsis admitted to a single ICU in Germany. Total number of patients screened not reported	Not reported	Within 24 hrs of diagnosis	125/154 (81 %)
Arabi [17] (2013)	2882 (33 %) patients with BMIs recorded from 8670 patients admitted with septic shock to 28 ICUs in Canada, Saudi Arabia, and the USA	Based on recorded weight and height	At ICU admission	2882/8670 (33 %)
Wacharasint [16] (2013)	730 (94 %) patients with BMIs recorded from 778 patients with septic shock admitted to 27 ICUs in Australia, Canada and the USA. A total of 6229 patients were screened for the trial and 802 were randomized. Number of patients with septic shock not enrolled is not noted	Based on recorded weight and height	At study enrollment for septic shock	730/778 (94 %)
Sakr [33] (2015)	2696 patients with sepsis from 8829 patients with BMIs recorded in a worldwide audit of 10,069 patients admitted to 730 ICUs in 84 countries. The total number of patients with sepsis out of all 10,069 patients was not reported. Sepsis was defined as infection with organ failure	Based either on recorded weight and height or on provider’s clinical estimate	Before onset of critical illness or at hospital admission	8829/10,069 (88 %)

*BMI* body mass index, *ICU* intensive care unit

Four studies adjusted the effect of BMI on mortality using age [15, 17, 32, 33], five adjusted for gender and comorbid illnesses [15–17, 32, 33], and all six adjusted for severity of acute illness with either Simplified Acute Physiology Score (SAPS) II or Acute Physiology and Chronic Health Evaluation (APACHE) II scores (Table 2). Other variables examined in each study are noted in Table 2. Three studies reported the effect of BMI on the adjusted odds ratio (aOR) of mortality [15, 17, 31]. Three studies reported adjusted hazards ratios, which were converted to aORs of mortality as described in the materials and methods section [16, 32, 33].

**Fig. 2:**
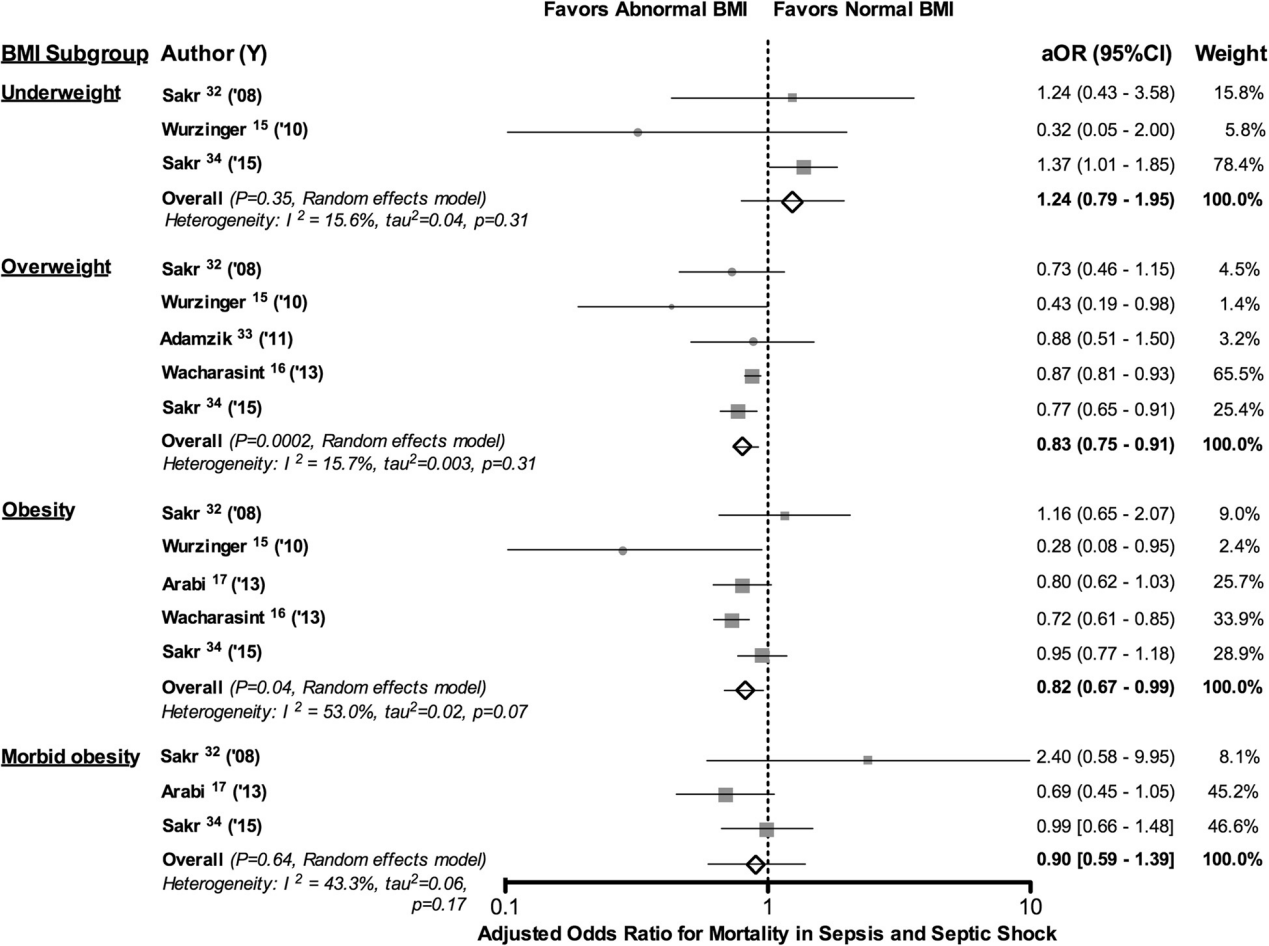
This figure shows the adjusted effects of different BMI categories on the odds ratio of mortality reported in studies examining patients requiring intensive care unit admission for sepsis, severe sepsis, or septic shock. Effects for underweight (<18.5 kg/m^2^), overweight (25 to <30 kg/m^2^), obese (30 to <40 kg/m^2^), and morbidly obese (≥40 kg/m^2^) BMIs were calculated compared to patients with normal BMIs (18.5 to 25 kg/m^2^ or <25 kg/m^2^). See the results section and Table 2 for variables that each study employed to adjust the effects of BMI on mortality as well as the definitions used for sepsis, severe sepsis, and septic shock

When stratified by BMI category, compared to a normal BMI (18.5 to 25 kg/m^2^) overweight (25 to <30 kg/m^2^) and obese (30 to <40 kg/m^2^) BMIs across five studies, each were associated with decreases in mortality that were significant [aOR: 0.83 (0.75, 0.91), *p* = 0.0002] and [aOR: 0.82 (0.67, 0.99), *p* = 0.04, respectively] (Fig. 2). These effects appeared consistent with low heterogeneity across studies examining overweight BMIs (I^2^ = 15.7 %, *p* = 0.31) but were less consistent with moderate heterogeneity across studies of obese BMIs (I^2^ = 53 %, *p* = 0.07). To strengthen our ability to detect an effect of moderately increased BMI on adjusted mortality, we combined obese BMI data from five studies with overweight BMI data from the one study without obese patients. Across these six patient groups (five obese and one overweight), the effects of increased BMI on reducing mortality were significant [aOR: 0.82 (0.69, 0.97), *p* = 0.02] and but had moderate heterogeneity (I^2^ = 42 %, *p* = 0.13). Over three studies each, morbidly obese (≥40 kg/m2) and underweight (<18.5 kg/m2) BMIs were not significantly associated with mortality [aOR: 0.90 (0.59, 1.39), *p* = 0.64; I^2^ = 43.3 %, *p* = 0.17 and aOR: 1.24 (0.79, 1.95), *p* = 0.35; I^2^ = 15.6 %, *p* = 0.31, respectively].

Risk of bias of each study was assessed using the Newcastle-Ottawa quality assessment scale for cohort studies (Additional file 4). Publication bias was to be assessed by funnel plot and Egger’s regression if sufficient data was available [34]. Because only three to five studies were present in each BMI subgroup, publication bias could not be assessed.

## Discussion

Different from our hypothesis, in studies of adults admitted to the ICU with sepsis, severe sepsis, or septic shock and which adjusted for other baseline variables, patients with overweight or obese BMIs, but not with morbidly obese ones, had reductions in mortality at up to 60 days compared to those with normal BMIs. There are several plausible biologic and physiologic reasons for these mortality reductions with the two former categories. First, increased adipose tissue is associated with increased renin-angiotensin system activity [35]. While this increased activity contributes to the hypertension of overweight and obese patients, it could also have protective hemodynamic effects during sepsis and decreased the need for fluid or vasopressor support, therapies which in excess can adversely impact outcome [36]. Second, increased lipoprotein levels and adipose tissue in patients with increased BMI may bind and inactivate lipopolysaccharide or other harmful bacterial products released during sepsis [37, 38]. Third, excess adipose tissue could provide increased beneficial energy stores during the catabolic septic state [39]. Finally, excess adipose tissue may have beneficial immune functions. For example, adipose tissue has been associated with increased production of both tumor necrosis factor (TNF) and soluble TNF receptor [40, 41]. While increased TNF production might augment protective host defense mechanisms during infection, increased soluble TNF receptor levels could reduce the deleterious effects of excessive TNF production during sepsis. Studies have suggested that obesity suppresses injurious inflammatory mediator release during sepsis and sepsis-associated acute lung injury [42].

Although morbidly obese BMIs were not associated with reductions in mortality, they, perhaps surprisingly, were also not associated with mortality increases. However, there were few studies and considerably fewer patients with morbidly obese BMIs investigated, and this may have limited our ability to demonstrate a potential survival benefit with this category. Also, as discussed further below, there may be a diminishing benefit as BMI levels exceed the overweight and obese categories.

It is possible that methodology in studies may have contributed to an apparent but not real reduction in mortality in septic patients with overweight and obese BMIs. First, mortality may not have been adjusted for baseline variables favoring improved outcomes in overweight and obese patients. While septic patients with increased BMI may have infections (e.g., skin and soft tissue) more responsive to treatment than those with normal BMIs, only two studies adjusted for the site and type of underlying infection [16, 17]. Only one study adjusted for interventions patients received at admission [15]. However, time to antibiotic therapy may have differed across BMI categories and impacted outcomes. Also, administration of non-weight-based therapies such as fluids or vasopressors (e.g., norepinephrine) may have benefited patients with increased BMIs. In two studies analyzed here, when weight was accounted for, septic patients with increased BMI received less overall fluid than normal weight patients and this may have protected organ function [16, 17]. Second, selection bias may have altered the results. Concerns about airway protection and hypoventilation in patients with increased BMI may have prompted intubation with mechanical ventilation and ICU admission in patients with more easily treated infection [43, 44]. Inability to administer adequate care for obese patients on general wards may have also caused ICU admission of obese patients with less severe infection [17]. Third, missing data may have influenced the results. The study with the largest number of septic patients did not provide adjusted mortality rates for those with underweight or overweight BMIs, even after our attempts to obtain this information from investigators. Finally, inaccurate height and weight estimates are common in the ICU setting and may have caused assignment of patients to incorrect BMI categories [45–47]. Weight first measured in the ICU following aggressive emergency room fluid resuscitation may have resulted in patients with normal BMIs at baseline being categorized as overweight or obese at the time of study entry [17]. Notably, only one study appeared to clearly define both how and when height and weight measurements were calculated [16], and no study explicitly reported the reliability of the BMI calculations made.

There are several potential limitations to this study. The most important one has to do with the design and selection of patients included in the studies analyzed. Four of the six studies were retrospective ones, with three of these studies having 87 % of the patients available to examine the influence of BMI on mortality in sepsis. The largest study, a retrospective one, only included 33 % of patients from the population available for analysis due to missing BMI data [17]. This study reported that included patients were significantly different from excluded patients, having fewer comorbid conditions but higher hospital mortality rates (Table 3) [17]. The second-largest study did not report the proportion of patients with sepsis that did not have a recorded BMI [33]. Those with unrecorded BMIs may have had fulminant sepsis and died prior to BMI measurement. This study also examined critical care patients admitted in 84 different countries over a 2-week period during the year. Clinical practice in intensive care units may have differed across centers and countries, while severity of illness and outcomes may have differed with seasonal variation [33]. The third-largest study was a retrospective analysis of a randomized controlled study of vasopressin therapy that included only 15 % of patients screened for enrollment (Table 3) [16]. However these three retrospective studies together contributed 90 % and 89 % respectively to the weight of the analyses suggesting that overweight or obese BMIs reduced adjusted mortality.

Other potential limitations include the following. First, the studies we examined which adjusted for comorbid illnesses that have been associated with increased BMI (e.g., diabetes, coronary artery disease) may have decreased or negated the potential detrimental effects of overweight or obese BMIs [48, 49]. Second, BMI does not differentiate changes in adipose versus muscle tissue. As people age, decreased BMI related to loss of muscle tissue (sarcopenia) [50–52] may be associated with a worsened outcome from sepsis. However, while BMI is not a perfect measure of adiposity [53], routine use of other measures of adiposity such as waist circumference, calipers or computed tomography, are not routinely performed in the ICU. Studies in our review likely utilized BMI as a measure of adiposity, due to its ease of use and measurement. Third, the studies included in this meta-analysis did not describe the types of nutritional support patients were receiving (e.g., low-calorie, high-protein therapy, conventional therapy, or nothing). Differences in nutritional support may have influenced outcome [54]. Finally, all the studies included in this analysis used sepsis definitions that predate the new sepsis-3 guideline nomenclature [55]. Five of the six studies used definitions of severe sepsis and septic shock by Bone et al. [25], and one study used a definition of sepsis by Vincent et al. [26]. As previously noted though, correlations can be drawn between this prior sepsis nomenclature and that of the new sepsis-3 guideline [55].

Despite its potential limitations, this study contributes in several ways to the current literature regarding the influence of increased BMI on outcomes in critically ill patients. With regard to sepsis specifically, a previous systematic review by Trivedi et al. [12] examined outcomes for obese and non-obese BMIs in adults and children admitted with sepsis to both ICU and non-ICU settings but did not incorporate a meta-analysis. Our systematic review has focused only on adult patients requiring ICU admission and has included a meta-analysis. In contrast to the prior review, the present one has also analyzed patients with morbidly obese BMIs and, in employing adjusted outcomes, has highlighted variables future analyses may need to consider (Table 2). Notably, our study and this prior one are in agreement regarding the need for more rigorous investigation into the potential impact of obesity on outcomes in septic patients.

With regard to the potential relationship between increased BMI and outcomes in critically ill patients in general, the present analysis adds to others suggesting that there may be an association between overweight and obese BMIs and unexpected increases in survival [56–58]. This possible relationship has been referred to as the obesity survival paradox since the documented adverse effects of obesity on chronic disease and long-term mortality would reasonably be expected to also worsen and not improve outcomes during acute illness of whatever nature [55]. However, the actual existence and basis for this apparent paradox are debated [8, 59, 60]. More in keeping with expectations, in critically ill populations as in the septic ones analyzed here, patients with underweight or morbidly obese BMIs have demonstrated either no increased survival or worsened survival [61–63]. This has lead to the proposal that the relationship between BMI and outcome during acute disease is U-shaped, with worsened outcomes only apparent at the extremes of increased or decreased BMIs [8, 60, 64]. Whether such a relationship clearly holds for patients with sepsis requires further investigation in well-designed studies that adequately adjust for other confounding conditions and variables.

## Conclusions

Obesity is a rapidly growing problem in the developed world, and determining whether it influences the outcome of critically ill patients with sepsis is important for both therapeutic and prognostic reasons. In this systematic review and meta-analysis of studies of adult patients requiring ICU care, adjusted mortality was reduced with overweight and obese BMIs in patients with sepsis, severe sepsis, and septic shock. However, while the present meta-analysis focused on studies reporting the effects of BMI adjusted for other influential baseline variables, questions regarding study design, patient selection, and BMI measurements make these findings difficult to interpret. Large, prospective studies, employing timely and validated measures of BMI, as well as case or propensity controlled study designs which adjust for multiple potential confounders, are necessary to better define the influence of increased BMI on sepsis ICU outcomes.

## Abbreviations

aHR, adjusted hazard ratio; aOR, adjusted odds ratio; APACHE, Acute Physiology and Chronic Health Evaluation; BMI, body mass index; ICU, intensive care unit; SAPS, Simplified Acute Physiology Score; SOFA score, Sepsis-related Organ Failure Assessment score; TNF, tumor necrosis factor

## Additional files

Additional file 1: Database search strategies. (DOC 31 kb) Additional file 2: Inclusion criteria. (DOC 27 kb) Additional file 3: Standardized protocol for data extraction. (DOC 27 kb) Additional file 4: Newcastle-Ottawa quality assessment scale for cohort studies. (DOC 36 kb) Additional file 5: PRISMA 2009 checklist. (DOC 66 kb)
