# Supplementary material for: Increased body mass index and adjusted mortality in ICU patients with sepsis or septic shock: a systematic review and meta-analysis
Source: Crit Care. 2016 Jun 15;20:181. doi: 10.1186/s13054-016-1360-z (PMC4908772; doi:10.1186/s13054-016-1360-z)
Supplement: Additional file 1: — Database search strategies. (DOC 31 kb) [file 13054_2016_1360_MOESM1_ESM.doc]

**Additional file 1: Database search strategies**

**1. MEDLINE**

**2. EMBASE
3. Cochrane Central Register of Controlled Trials (CENTRAL)**

**1. MEDLINE**

(sepsis[majr] OR sepsis[tiab] OR septic[tiab] OR "septic shock"[tiab] OR "bacterial infections and mycoses"[majr] OR infection[tiab] OR infections[tiab] OR "virus diseases"[majr] OR pneumonia[majr] OR pneumonia[tiab] OR "influenza, human"[majr] OR influenza[tiab] OR "acute lung injury"[majr] OR "acute lung injury"[tiab] OR "respiratory distress syndrome, adult"[majr] OR "adult respiratory distress syndrome"[tiab] OR "acute respiratory distress syndrome"[tiab]) AND (obesity[majr] OR obesity[tiab] OR obese[tiab] OR overweight[majr] OR overweight[tiab] OR "body mass index"[majr] OR "body mass index"[tiab] OR BMI[tiab] OR "bariatric surgery"[majr] OR bariatric[tiab]) AND ("epidemiologic study characteristics as topic"[mesh] OR "epidemiologic factors"[mesh] OR comorbid[tiab] OR comorbidity[tiab] OR "regression analysis"[mesh] OR regression[tiab] OR multivariate[tiab] OR "analysis of variance"[mesh] OR "meta-analysis as topic"[mesh] OR "meta-analysis"[pt] OR "meta-analysis"[tiab] OR "meta-analyses"[tiab] OR "clinical trials as topic"[mesh] OR "clinical trial"[pt] OR "clinical trial"[tiab] OR "clinical trials"[tiab] OR "randomized controlled trial"[pt] OR "randomized controlled trial"[tiab] OR "randomized controlled trials"[tiab] OR observation[mesh] OR "observational study"[pt] OR "observational study"[tiab] OR "observational studies"[tiab] OR "comparative study"[pt] OR "comparative study"[tiab] OR "comparative studies"[tiab] OR "systematic"[sb] OR "systematic review"[tiab] OR "systematic reviews"[tiab])

**2. EMBASE**

(sepsis:ti OR septic:ti OR 'septic shock':ti OR 'infection'/exp/mj OR infection:ti OR infections:ti OR 'pneumonia'/exp/mj OR pneumonia:ti OR influenza:ti OR 'respiratory distress syndrome'/exp/mj OR 'acute lung injury':ti OR 'adult respiratory distress syndrome':ti OR 'acute respiratory distress syndrome':ti) AND ('obesity'/exp/mj OR obesity:ti OR obese:ti OR overweight:ti OR 'body mass'/exp/mj OR 'body mass index':ti OR bmi:ti OR 'bariatric surgery'/exp/mj OR bariatric:ti)

**3. Cochrane Central Register of Controlled Trials (CENTRAL)**

ID            Search  Hits

#1           sepsis:ti                1219

#2           septic:ti                722

#3           [mh sepsis]         3068

#4           infection:ti          14032

#5           infections:ti        14032

#6           [mh "bacterial infections and mycoses"]               28326

#7           [mh "virus diseases"]     19225

#8           pneumonia:ti     2163

#9           [mh pneumonia]              2577

#10         influenza:ti         2219

#11         [mh "influenza, human"]              1333

#12         "acute lung injury":ti      262

#13         [mh "acute lung injury"]               114

#14         "adult respiratory distress syndrome":ti                68

#15         "acute respiratory distress syndrome":ti               337

#16         [mh "respiratory distress syndrome, adult"]        600

#17         #1 or #2 or #3 or #4 or #5 or #6 or #7 or #8 or #9 or #10 or #11 or #12 or #13 or #14 or #15 or #16                 54478

#18         obesity:ti             2335

#19         obese:ti               3693

#20         [mh obesity]      7599

#21         overweight:ti     1821

#22         [mh overweight]              8277

#23         "body mass index":ti      370

#24         [mh "body mass index"]               6061

#25         BMI:ti    112

#26         bariatric:ti            268

#27         [mh "bariatric surgery"]                764

#28         #18 or #19 or #20 or #21 or #22 or #23 or #24 or #25 or #26 or #27              14503

#29         #17 and #28        203

Limited to TRIALS             187 records
