# Supplementary material for: Increased body mass index and adjusted mortality in ICU patients with sepsis or septic shock: a systematic review and meta-analysis
Source: Crit Care. 2016 Jun 15;20:181. doi: 10.1186/s13054-016-1360-z (PMC4908772; doi:10.1186/s13054-016-1360-z)
Supplement: Additional file 2: — Inclusion criteria. (DOC 27 kb) [file 13054_2016_1360_MOESM2_ESM.doc]

**Additional file 2: Inclusion Criteria**

- Original research
- All patients are adults (at least 16 years old)
- All patients admitted to intensive care unit
- All patients had sepsis, severe sepsis, or septic shock
- All patients had a measure of body mass index or obesity/ morbid obesity was defined
- Presence of outcome (mortality)
  - 1. Intensive care unit mortality
    2. Hospital mortality
    3. 30-day mortality
    4. 60-day mortality
    5. 180-day mortality
- Multivariate analyses performed with outcome (mortality) adjusted for potential confounders, such as age, sex, co-morbid illnesses or severity of illness
