# Supplementary material for: Increased body mass index and adjusted mortality in ICU patients with sepsis or septic shock: a systematic review and meta-analysis
Source: Crit Care. 2016 Jun 15;20:181. doi: 10.1186/s13054-016-1360-z (PMC4908772; doi:10.1186/s13054-016-1360-z)
Supplement: Additional file 3: — Standardized protocol for data extraction. (DOC 27 kb) [file 13054_2016_1360_MOESM3_ESM.doc]

**Additional file 3: Standardized protocol for data extraction**

- Name of first author
- Publication year
- Country
- Study design
- Study period
- Study definition and diagnostic criteria of sepsis, severe sepsis or septic shock
- Mean age of study population
- Male percentage
- Presence of outcome (mortality)
  - Intensive care unit mortality
  - Hospital mortality
  - 30-day mortality
  - 60-day mortality
  - 180-day mortality
- Adjusted odds ratio for mortality (95% confidence interval)
- Sample size
- Body mass index categories and definitions
- Non-survivors as a proportion of total sample
- Variables included in multivariate analysis to adjust for potential confounding
