# Supplementary material for: Increased body mass index and adjusted mortality in ICU patients with sepsis or septic shock: a systematic review and meta-analysis
Source: Crit Care. 2016 Jun 15;20:181. doi: 10.1186/s13054-016-1360-z (PMC4908772; doi:10.1186/s13054-016-1360-z)
Supplement: Additional file 4: — Newcastle-Ottawa quality assessment scale for cohort studies. (DOC 36 kb) [file 13054_2016_1360_MOESM4_ESM.doc]

**Additional file 4: Newcastle-Ottawa Quality Assessment Scale for Cohort Studies**

**[Note: A study can be awarded a maximum of one star for each numbered item within the Selection and Outcome categories. A maximum of two stars can be given for Comparability](http://www.ohri.ca/programs/clinical_epidemiology/nosgen.pdf#page=1)**

**A. Selection**

**1) Representativeness of the exposed cohort**

a) Truly representative of the average cohort of patients with sepsis *

b) Somewhat representative of the average cohort of patients with sepsis *

c) Selected group of users e.g. nurses, volunteers

d) No description of the derivation of the cohort

**2) Selection of the non-exposed cohort**

a) Drawn from the same community as the exposed cohort *

b) Drawn from a different source

c) No description of the derivation of the non-exposed cohort

**3) Ascertainment of exposure**

a) Secure record (e.g. medical records) *

b) Structured interview *

c) Written self report

d) No description

**4) Demonstration that outcome of interest was not present at start of study**

a) Yes *

b) No

**B. Comparability**

**1) Comparability of cohorts on the basis of the design or analysis**

a) Study controls for severity of illness *

b) Study controls for ALL of the following: age, co-morbidity, and site of infection *

**C. Outcome**

**1) Assessment of outcome**

a) Independent blind assessment *

b) Record linkage *

c) Self report

d) No description

**2) Was follow-up long enough for outcomes to occur**

a) Yes (ICU stay, hospital stay, 28d, 30d and 60d considered adequate follow up period for outcome of interest) *

b) No

**3) Adequacy of follow up of cohorts**

a) Complete follow up - all subjects accounted for *

b) Subjects lost to follow up unlikely to introduce bias; small number lost to follow-up (> 90 % follow up), or description provided of those lost) *

c) Follow up rate < 90% and no description of those lost

d) No statement

|  | **A. Selection** | | | | **B. Comparability of cohorts** | **C. Outcome** | | |
| --- | --- | --- | --- | --- | --- | --- | --- | --- |
|  | Represent- ativeness of exposed cohort | Selection of non-exposed | Ascertainment of Exposure | Outcome not present at start |  | Assessment of exposure | F/U long enough? | Adequacy of F/U |
| Sakr  2008 | ***** | ***** | ***** | ***** |  | ***** | ***** |  |
| Adamzik  2011 | ***** | ***** | ***** | ***** | ***** |  | ***** | ***** |
| Arabi  2013 |  | ***** | ***** | ***** | ****** | ***** | ***** |  |
| Wurzinger  2010 | ***** | ***** | ***** | ***** | ****** | ***** | ***** | ***** |
| Wacharasint  2013 | ***** | ***** | ***** | ***** | ****** | ***** | ***** | ***** |
| Sakr  2015 | ***** | ***** | ***** | ***** |  | ***** | ***** |  |
